# The Reprimo Gene Family: A Novel Gene Lineage in Gastric Cancer with Tumor Suppressive Properties

**DOI:** 10.3390/ijms19071862

**Published:** 2018-06-25

**Authors:** Julio D. Amigo, Juan C. Opazo, Roddy Jorquera, Ignacio A. Wichmann, Benjamin A. Garcia-Bloj, Maria Alejandra Alarcon, Gareth I. Owen, Alejandro H. Corvalán

**Affiliations:** 1 Departamento de Fisiología, Facultad de Ciencias Biológicas, Pontificia Universidad Católica de Chile, 8330025 Santiago, Chile; jamigo@bio.puc.cl (J.D.A.); gowen@bio.puc.cl (G.I.O.); 2 Instituto de Ciencias Ambientales y Evolutivas, Facultad de Ciencias, Universidad Austral de Chile, 5090000 Valdivia, Chile; jopazo@gmail.com; 3 Laboratory of Oncology, Facultad de Medicina, Pontificia Universidad Católica de Chile, 8330034 Santiago, Chile; ignacio.wichmann@gmail.com (I.A.W.); garciabloj@gmail.com (B.A.G.-B.); mralarco@uc.cl (M.A.A.); 4 Departamento de Oncología y Hematología, Facultad de Medicina, Pontificia Universidad Católica de Chile, 8330034 Santiago, Chile; 5 CORE Biodata, Advanced Center for Chronic Diseases (ACCDiS), Pontificia Universidad Católica de Chile, 8330024 Santiago, Chile; roddy.jorquera@gmail.com; 6 Millennium Institute on Immunology and Immunotherapy, Pontificia Universidad Católica de Chile, 8331150 Santiago, Chile

**Keywords:** gastric cancer, reprimo, tumor suppressive gene properties, development, evolution, biomarker

## Abstract

The reprimo (*RPRM*) gene family is a group of single exon genes present exclusively within the vertebrate lineage. Two out of three members of this family are present in humans: *RPRM* and *RPRM-Like* (*RPRML*). *RPRM* induces cell cycle arrest at G2/M in response to p53 expression. Loss-of-expression of *RPRM* is related to increased cell proliferation and growth in gastric cancer. This evidence suggests that *RPRM* has tumor suppressive properties. However, the molecular mechanisms and signaling partners by which *RPRM* exerts its functions remain unknown. Moreover, scarce studies have attempted to characterize *RPRML*, and its functionality is unclear. Herein, we highlight the role of the *RPRM* gene family in gastric carcinogenesis, as well as its potential applications in clinical settings. In addition, we summarize the current knowledge on the phylogeny and expression patterns of this family of genes in embryonic zebrafish and adult humans. Strikingly, in both species, *RPRM* is expressed primarily in the digestive tract, blood vessels and central nervous system, supporting the use of zebrafish for further functional characterization of *RPRM*. Finally, drawing on embryonic and adult expression patterns, we address the potential relevance of *RPRM* and *RPRML* in cancer. Active investigation or analytical research in the coming years should contribute to novel translational applications of this poorly understood gene family as potential biomarkers and development of novel cancer therapies.

## 1. Introduction

The reprimo (*RPRM*) gene family is a novel and poorly understood single-exon intronless gene family. *RPRM* genes are expressed across many species and are associated with developmental patterning of the gastrointestinal tract, brain and blood vessels [1,2]. In humans, who have only *RPRM* and *RPRM-Like* (*RPRML)*, the *RPRM* gene appears to be involved in tumor suppression in tumors of the gastrointestinal tract as well as in multiple other organs. Expression of *RPRM* is mainly induced by p53 after DNA damage, though expression of this gene has also been associated to expression of p73, another member of the *p53* gene family [3]. Upregulation of RPRM results in cell cycle arrest at the G2/M checkpoint [4]. Epigenetic silencing of *RPRM*, mainly by DNA methylation of its promoter region, occurs at early stages of human cancer. Assessment of this biochemical DNA modification in body fluids has opened an interesting opportunity for translational applications of this family of genes, as cancer biomarkers.

Here, we describe and discuss the structure, genomic location and homologies of these poorly characterized genes. In addition, we explore the role in development as well as functional diversification of the *RPRM* family. In gastric cancer as well as in other human neoplasms, this family of single exon and intronless genes had led to the discovery of novel clinical applications such as non-invasive biomarkers for early diagnosis and disease monitoring and the development of new drugs for cancer therapies.

## 2. Structure and Genomic Location of the *RPRM* Gene Family

The *RPRM* gene family is composed of three members: *RPRM*, *RPRML* and *RPRM3*. While *RPRM* and *RPRML* are expressed in most of the vertebrate lineages, *RPRM3* is only found in cartilaginous fish (e.g., sharks and rays), bony fish (e.g., zebrafish) and coelacanths [1]. In humans, *RPRM* and *RPRML* are both single exon and intronless genes, which is an uncommon type of gene representing roughly 3% of the human genome [5]. *RPRM* and *RPRML* are located on the minus strand of chromosomes 2q23.3 and 17q21.32, respectively. *RPRM* spans 1.47 kb of genomic DNA and encodes a 109-amino acid protein with an estimated molecular weight of 11,774 Da. Similarly, *RPRML* spans 1.09 kb of genomic DNA encoding a 120-amino acid protein with an estimated molecular weight of 12,312 Da. *RPRM3* is also a single exon gene and intronless gene, that is located on the minus strand of chromosome 23q32.2 in zebrafish, *RPRM3* spans 1.794 kb of genomic DNA and encodes a 103-amino acid protein with an estimated molecular weight of 11,323 Da.

**Figure 1:**
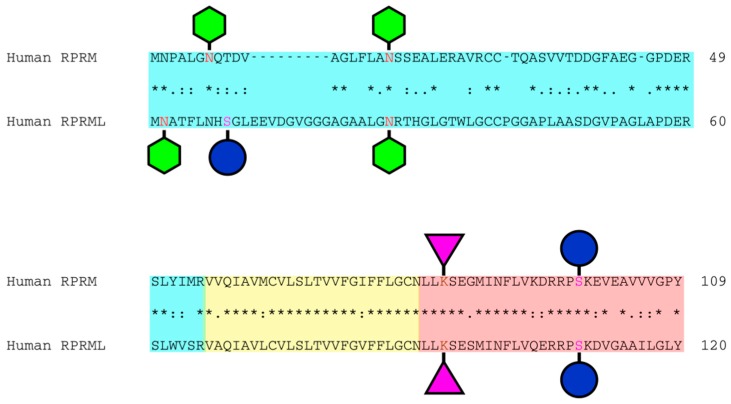

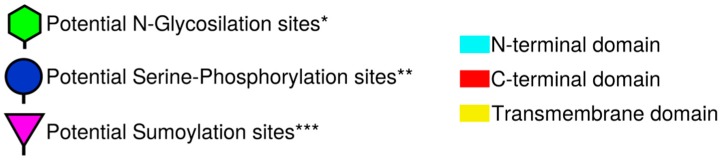
Domain structure and potential post-translational modification sites of human RPRM and RPRML proteins. Schematic representation shows the RPRM and RPRML putative *N*-glycosylation (green hexagons), serine-phosphorylation (blue circle) and sumoylation (purple triangle) sites. The N-terminal, transmembrane and C-terminal domains are represented by colored boxes (turquoise, yellow, and red, respectively). An * (asterisk) indicates positions which have a single, fully conserved residue. A: (colon) indicates conservation between groups of strongly similar properties—scoring >0.5 in the Gonnet PAM 250 matrix. A. (period) indicates conservation between groups of weakly similar properties—scoring 0.5 in the Gonnet PAM 250 matrix. Post-translational modification sites were predicted using *NetNGlyc (http://www.cbs.dtu.dk/services/NetNGlyc/), **NetPhos (http://www.cbs.dtu.dk/services/NetPhos/) and ***SumoPlot (http://www.abgent.com/sumoplot).

RPRM is a highly glycosylated protein which has two *N*-glycosylation sites at amino acids 7 and 18, a potential serine-phosphorylation site at residue 98, a predicted sumoylation site at position 82 and a potential transmembrane domain covering amino acids 56 to 76 (Figure 1) [6]. Furthermore, *RPRML* has predicted *N*-glycosylation sites at amino acids 2 and 27, a predicted sumoylation site at position 93 and a transmembrane site covering amino acids 67 to 87 (Figure 1). As other intronless gene families, such as *JUN* and *FOX*, the *RPRM* gene family is often implicated in cancer through their overrepresentation in cell growth and proliferation [7]. Functional analyses have suggested that *RPRM* is a transcriptional target for p53 and as a cell cycle arrest protein at the G2/M checkpoint, operating through inhibition of the nuclear translocation of the Cdc2/cyclin B1 complex [4].

## 3. Evolution of the *RPRM* Gene Family

**Figure 2:**
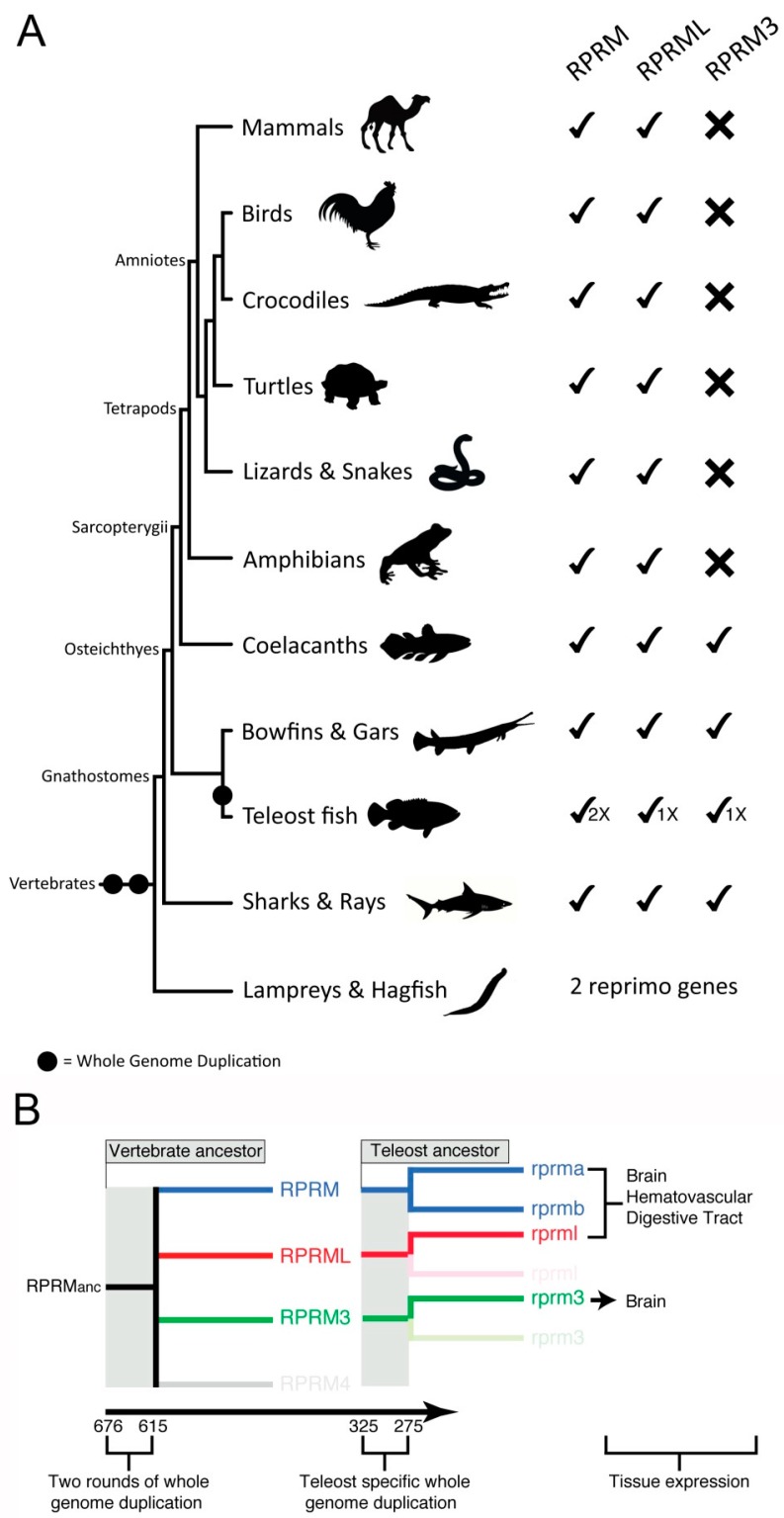
Evolution and diversification of *RPRM* genes in vertebrates. (**A**) During the diversification of vertebrates *RPRM* genes were differentially retained, as not all *RPRM* genes are present in all main groups of vertebrates. Thus, *RPRM* and *RPRML* genes were retained in all main groups of jawed vertebrates (gnathostomes), whereas *RPRM3* was only retained in four distantly related groups: cartilaginous fish (e.g., sharks, skates, and rays), holostean fish (e.g., bowfish and gars), teleost fish (e.g., zebrafish) and coelacanths. In the group that includes lampreys and hagfish, two *RPRM* genes have been identified, however, phylogenetic and synteny analyses have failed in defining orthology. (**B**) Schematic representation of the evolution of the *RPRM* gene family in teleost fish. On the left, the *RPRM* gene family diversified as a product of the two rounds of whole-genome duplication occurred in the vertebrate ancestor, as well as, a teleost-specific whole genome duplication. On the left, the expression territories of the three *RPRM* gene lineages present in teleost fish.

Based on current sequenced genome data, the evolutionary history of the *RPRM* gene family traces back to the last common ancestor of vertebrates, which lived between 676 and 615 million of years ago [1]. At that time the ancestor of vertebrates presumably had only a single *RPRM* gene in its genome, which likely performed many of the physiological functions that the current gene family performs today. Through time, this ancestral gene diversified as a consequence of the two rounds of whole genome duplications (WGDs), which occurred early in the evolutionary history of vertebrates [8,9]. Thus, the single copy gene present in the genome of the vertebrate ancestor gave rise to four *RPRM* genes [1], three of which were retained in actual vertebrates (Figure 2A). The presence of co-duplicated genes that are found on the chromosomes where the *RPRM* genes are located in actual species, and the fact that the genomic regions in which the *RPRM* genes are located derived from a single linkage group in the chordate ancestor support this hypothesis [10].

During the diversification of vertebrates the *RPRM* gene family was differentially retained, as not all *RPRM* genes are present in all main vertebrate groups (Figure 2A). Although it was previously claimed that the *RPRM* gene was not retained in birds [1], searches in the most recent version of bird genomes revealed the presence of the *RPRM* gene in this subgroup of amniotes. Thus, all groups of jawed vertebrates (gnathostomes) have retained this gene (Figure 2A). The *RPRML* gene has also been identified in all main groups of gnathostomes, whereas *RPRM3* was only retained in four distantly related vertebrate groups: cartilaginous fish (e.g., sharks, skates, and rays), holostean fish (e.g., bowfish, gars), teleost fish (e.g., zebrafish) and coelacanths (Figure 2A). In cyclostomes, the group that includes lampreys and hagfish, two *RPRM* genes has been identified (Figure 2A). However, phylogenetic reconstructions have failed in defining orthology. *RPRM* sequences of this group are recovered sister to each other, which might be due to features of their genomes (e.g., GC bias) that differ substantially from all other vertebrate genomes [11,12,13], making it difficult to recover the true evolutionary history using phylogenetic approaches. 

More recently in vertebrate history, the *RPRM* gene family further expanded as a consequence of the teleost-specific genome duplication (TSGD) that occurred in the ancestor of this group between 325 and 275 million of years ago [14,15] (Figure 2A). Although the TSGD potentially doubled the number of all *RPRM* genes in the common ancestor of teleosts, only the *rprm* gene retained duplicated copies, termed *rprma* and *rprmb* (Figure 2A). 

## 4. Developmental Expression Patterns of *RPRM* Gene Family

The use of animal models is the primary step to understand the process of human carcinogenesis and the development of new drugs for cancer therapies [16]. In this scenario, Zebrafish combines the complexity of a whole vertebrate animal with the easy-to-use and high-throughput characteristics of in vitro models [17,18]. Despite differences with teleosts, most of the genetic pathways that regulate development are similar between zebrafish and human [19]. In zebrafish embryos—both duplicated copies of *rprm* (*rprma* and *rprmb*) are expressed in a tissue-specific manner in the gastrointestinal tract, brain and blood vessels. Strikingly, expression of RPRM protein in adult human was also detected in the same organs [2]. Expression of *rprm3*, a gene that is lost in all tetrapods, is restricted to the central nervous system (CNS) in zebrafish embryos and larvae [2].

Our recent observations also indicate that the three *rprm* genes (*rprma*, *rprmb* and *rprml*) genes are expressed in the developing digestive tract in embryonic fish [2]. Importantly, in humans, *RPRM* is a gene which expression is lost in many human gastrointestinal malignancies and serves as a potential biomarker for non-invasive detection of gastric cancer (reviewed in the following section). The RPRM protein is located in epithelial cells lining the intestinal crypts and gastric glands and smooth muscle cells (SMCs) from the *Muscularis propria* [2], As in humans, the digestive tube of the zebrafish is organized in concentric layers of SMCs [20]. However, the role of *RPRM* genes during the differentiation process of gastrointestinal epithelial cells, which occurs during the migration of these cells from the base of gastric glands and intestinal crypts, remains to be determined. In this scenario, the zebrafish model organism offers an interesting alternative to dissect this role in developmental physiology.

Figueroa et al. [2] also show that, in zebrafish and humans, the *RPRM* and *RPRML* genes are expressed in vascular tissues. In humans, RPRM is expressed in endothelial and vascular smooth muscle cells (VSMCs). The expression of RPRM and RPRML in blood vessels suggests a potential involvement of *RPRM* during angiogenesis or vasculogenesis. Future studies require molecular manipulation of the *RPRM* gene family to unveil the role of *RPRM* genes in the formation and dynamics of the blood vessels.

As we recently reported, in embryonic zebrafish larvae, *rprma*, *rprmb* and *rprml* are expressed distinctly in the developing brain [21]. Some of the structures that express these genes include the forebrain, telencephalon and the olfactory epithelium (OE), among other sensory organs. For example, the expression of *rprma* is largely restricted to the peripheral nervous system at the OE, while *rprmb* transcripts are located in most posterior neuronal territories such as the optic tectum and trigeminal ganglia. In contrast to *rprma* and *rprmb*, expression of *rprml* is not detectable in the retina. In adult zebrafish and humans, the RPRM messenger and protein are both located in brain tissues. The human RPRM is expressed in grey and white matter neurons and glial cells from the brain cortex, a tissue that displays low mitotic rates during adulthood [2,21,22]. These findings suggest that the *RPRM* genes may play a key role in the regulation of cell proliferation in brain development and/or regeneration during adulthood [21]. Models that compare brain-specific gene expression profiles between wild-type animals and those with loss-of-function of *rprm* and *rprml* will help to define the expression and function of the *RPRM* gene family in the processes that contribute to brain development. 

## 5. Functional Diversification of the *RPRM* Gene Family

Due to the three WGDs, the diversification of the *RPRM* gene family opens new opportunities for physiological innovation within this lineage. In the literature it has been difficult to probe the causal link between the WGDs and biological innovation [23], although for some well-studied gene families, this causal association has been demonstrated [24,25,26]. As mentioned above, expression profile analyses of the *RPRM* genes demonstrate that they exhibit unique—although partially overlapping—expression patterns during embryonic and larval vascular development [2]. On one hand, *rprma*, *rprmb* and *rprml* are all expressed in the digestive tube, blood vessels and brain; whereas *rprm3* possesses a unique expression profile restricted only to the brain. Importantly, the expression patterns of *rprma* and *rprmb* transcripts in the zebrafish resembled expression profiles of the RPRM protein in humans. This evidence suggests that the developmental expression pattern for the *RPRM* gene family is the same in fish and mammals [1]. Furthermore, the compartmentalization in humans of *RPRM* genes in partially overlapping territories seems to agree with the pattern described for teleost fish (Figure 2B) [27]. Future studies should elucidate the functional role of the *RPRM* gene family during the physiological processes such as gut, vascular and neuronal development across the vertebrate subphylum.

## 6. Role of the *RPRM* Gene Family in Human Carcinogenesis

**Figure 3:**
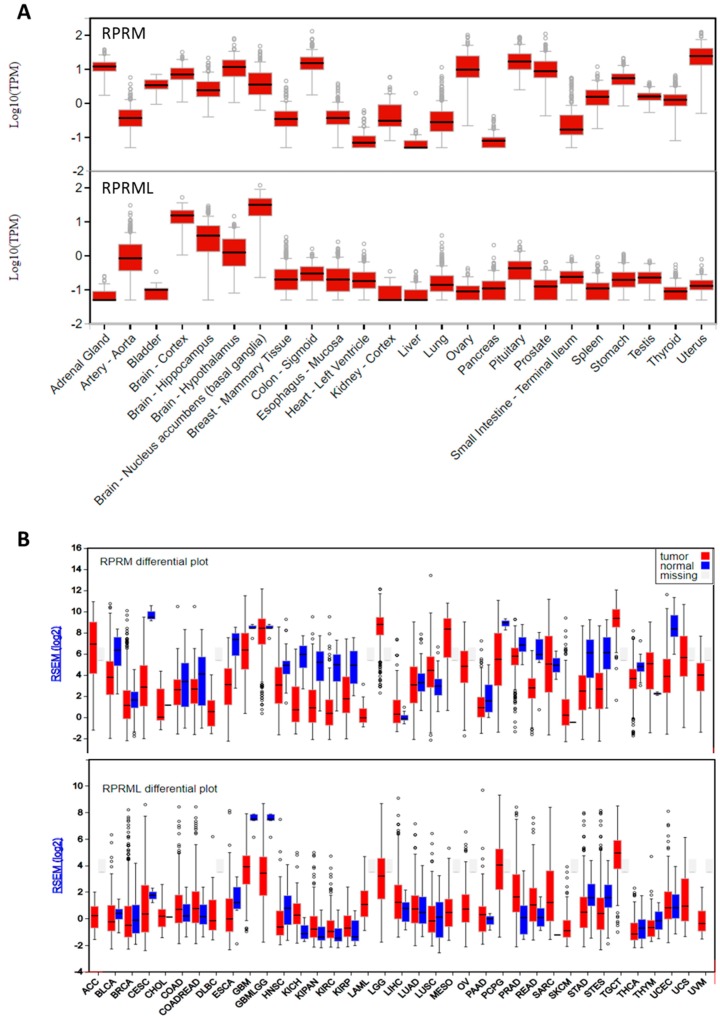
RNA expression of *RPRM* and *RPRML* across different tissues. (**A**) Tissue-specific expression profile from 570 human donors available in the Genotype-Tissue Expression (GTEx) database [28]. Data is expressed as log10 of Transcripts Per Kilobase Million (TPM). (**B**) Expression levels of human tumor and matched normal tissue samples from Broad Institute TCGA Genome Data Analysis Center [30]. Data is expressed as log2 of RSEM (RNA-Seq by Expectation Maximization).

In order to uncover the *RPRM* and *RPRML* tissue expression patterns in human samples, we have assessed in silico RNAseq data from the Genotype Tissue Expression (GTEx) database [28]. As shown in Figure 3A, *RPRM* has a variable expression across different tissues, whereas *RPRML* is expressed at very low levels in most tissues, except for brain compartments where expression levels are the highest. However, low transcript levels are expected for intronless genes, which generally express at lower levels than intron-containing genes despite having important biological roles [29]. Transcriptome studies in several vertebrate species reveal that *RPRM* genes are mainly expressed in the central nervous system. In accordance with this observation, our previous studies have shown that the transcript and protein for *RPRM* are expressed in the zebrafish and human brain [2]. In the case of cancer tissues, as shown in Figure 3B, both genes display down regulated expression in tumor tissues in comparison with non-tumor adjacent mucosa, including gastric cancer [30].

At experimental and clinical levels, only the *RPRM* gene has been examined in terms of biological functions and significance in human cancer. In gastric cancer cells, restoring the expression of *RPRM* by transfecting exogenous cDNA results in reduced colony formation and anchorage-independent growth [3,31]. Correspondingly, mouse xenografts models of gastric cancer cells deficient in *RPRM* expression have demonstrated enhanced tumor formation and volume [31,32]. In other tumors, such as breast cancer, pituitary tumors and renal cell carcinoma cell lines, overexpression of RPRM suppresses cell proliferation, cell migration, clonogenic capacity and invasiveness [33,34,35]. Furthermore, the role of RPRM in cell-cycle has also been explored. Ohki et al. [4] overexpressed RPRM through adenoviral infection in cells with wild-type (HeLa, Lovo, MCF7) and mutated (DLD1 and Saos2) p53, observing cell-cycle arrest in G2/M phase, independently of p53 mutational status. However, conflicting results have been reported in gastric cancer and pituitary cell lines where RPRM overexpression results in a significant increase in the sub-G1 population with minimal changes in S and G2/M populations [31,34]. Ectopic expression of RPRM cDNA in gastric cancer cell lines after exposure to DNA-damaging agents, such as 5-fluorouracil or cisplatin, results in an apoptotic phenotype 24 h after treatment [31]. In other types of tumors, with both wild-type and mutated *p53* gene, overexpression of RPRM induces an apoptotic phenotype after 4 days of adenoviral infection, suggesting that RPRM may also repress cell growth by induction of apoptosis [4]. Taken together, these results suggest that *RPRM* has tumor suppressive properties not only in gastric cancer but also in other tumors. 

Clinical studies have shown that the loss of *RPRM* expression is as common event in gastric cancer [3,31,32,36] as in other tumors of the gastrointestinal tract including Barrett’s-associated esophageal adenocarcinoma, pancreatic and colorectal carcinoma [37,38,39,40,41]. Loss of expression of *RPRM* has been also reported in non-digestive tumors including breast, renal cell carcinoma, adrenocortical and pituitary tumors [33,34,35,42,43]. Conversely, enhanced *RPRM* expression has been described in metastatic brain tumors [44].

*RPRM* is located within a CpG-enriched region of the genome. In these regions, a significant proportion of cytosines contain a methyl group in the fifth carbon when they are immediately preceded by a guanine (CpG sites). Although unevenly distributed across the genome, CpG sites generally cluster near gene promoters (CpG islands), thus controlling local chromatin structure and transcription factor binding [45]. In normal cells, a few CpG islands are usually methylated (DNA methylation), maintaining genomic stability and controlling expression of tissue-specific, imprinted and housekeeping genes [46]. In contrast, an aberrant pattern of DNA methylation has been observed in some cancers characterized by a genome wide low methylation state that promotes transcriptional activation of oncogenes, genomic instability, and loss of imprinting, while some CpG islands, particularly those located in the promoter regions of tumor suppressor genes, show a local hypermethylated state that may result in gene silencing [47]. This is one of the most common epigenetic alterations found in human cancers. In the case of *RPRM*, bisulfite sequence experiments to evaluate the density of methylated CpG sites of the promoter region have correlated positively with the levels of the transcriptional expression of the gene [3]. Consequently, restoring the expression of *RPRM* by the use of demethylating agents, such as 5-aza-cytidine has confirmed that the expression of *RPRM* gene is regulated by DNA methylation in gastric cancer cells [3,32,48]. In other neoplasm similar results have been obtain confirming the role of DNA methylation as the main mechanisms of regulation of *RPRM* gene expression [35,49]. In addition, DNA methylation of *RPRM* has been associated with a compact chromatin structure and further increasing transcriptional silencing of the gene [49]. Interestingly, an in agreement with the enhanced *RPRM* expression in metastatic brain tumors [44], bisulfite sequence studies in pituitary tumors have shown that loss of *RPRM* is not due to hypermethylation of the promoter region [34]. This observation raises the possibility that other mechanisms, genetic and/or epigenetic (i.e., microRNAs), might contribute to *RPRM* gene regulation. 

As previously mentioned, *RPRM* has been proposed as a transcriptional target for p53. In gastric cancer cells expressing wild-type p53, a significant down-regulation of RPRM has been described. Conversely, RPRM-induced changes were not seen in p53-deficient NCI-N87 cells. [50]. Analogous findings have been described by in silico analysis of the TCGA data, where a negative correlation between Survivin and RPRM expression was identified exclusively in patients with wild-type p53 protein status [50].

**Figure 4:**
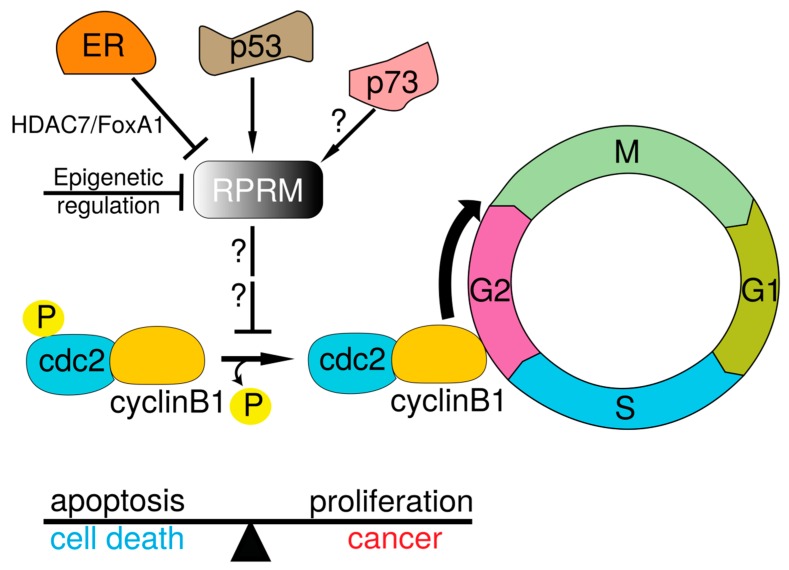
Schematic model of RPRM-mediated cell cycle and G2 arrest mechanisms. *RPRM* has been identified as a transcriptional target for: (1) p53 [4]; (2) histone deacetylase 7/FoxA1 (HDAC7/FoxA1) in an estrogen mediated mechanism [49]; and (3) for epigenetic silencing by hypermethylation of its promoter region [54]. A potential regulation by p73 it has also been proposed [3]. RPRM expression results in inhibited dephosphorylation of Cdc2, suppressing the activation of the Cdc2-Cyclin B1 complex. Thus, inducing cell cycle arrest at G2 suggesting a potential role for *RPRM* as a tumor suppressor gene [4]. The balance towards cell cycle arrest or proliferation can be shifted by multiple antagonistic effectors, amongst them *RPRM*. Straight lines with arrowheads indicate activation. Lines with no arrowhead indicate inhibition. Curved arrow on the bottom left indicates dephosphorylation of Cdc2/Cyclin B1 complex. Curved thick arrow on the bottom right indicates nuclear translocation of dephosphorylated Cdc2/Cyclin B1 at the G2/M checkpoint.

Based on a positive co-expression between RPRM and p73 proteins in a large cohort of tumor samples, the possibility that other members of the *p53* gene family participate in the regulation of *RPRM* has been raised [3]. Since cytoplasmic overexpression of RPRM inhibit nuclear translocation of the Cdc2-Cyclin/B1 complex inducing cell cycle arrest at the G2/M stage [4] further binding and or co-immunoprecipation experiments should contribute to clarify the role of p73 in the regulation of the expression of RPRM (Figure 4).

The clinical significance of the loss of RPRM expression in gastric cancer was first explored by Luo et al. [36]. This study analyzed RPRM protein expression along with tumor suppressor S100A2 (S100 calcium binding protein A2) in a cohort of 100 consecutive gastric cancer cases identifying loss of RPRM expression in up to 65% of cases. Interestingly, Luo et al. [36] found that there exists a positive relationship between the expressions of both genes. Furthermore, loss of RPRM expression was significantly associated with depth of tumor invasion, lymphatic vascular invasion and lymph node metastasis. These clinical findings have been confirmed by Saavedra et al. [3] showing also that loss of RPRM expression is particularly associated with the progression from stage I to stages III-IV (Japanese classification of gastric carcinoma) [51] in a cohort of Hispanic/Amerindian cases from Latin America, one of the highest regions in gastric cancer incidence worldwide [52]. Although none of the earlier studies were able to show that loss of expression of RPRM could influence overall survival in gastric cancer, our group has recently found that loss of RPRM expression does confer a worse prognosis only when accompanied with overexpression of Survivin, a well establish oncogene in gastric cancer [50,53]. Taken together, data suggest that *RPRM* requires a genetic background including other cancer-related genes, such as S100A2 and/or Survivin to drive the gastric carcinogenesis process.

Interestingly, it has recently been shown that upregulation of endogenous RPRM expression by the use of CRISPR/dCas9 (Clustered Regularly Interspaced Short Palindromic Repeats and associated dead Cas9) system, a platform that utilizes a catalytically deactivated Cas9 (dCas9) linked to effector domains for gene expression regulation (i.e., Synergistic Activation Mediator (SAM) complex) reduced cell proliferation and increased apoptosis in gastric cancer cells [55]. This finding has been expanded to other genes with tumor suppressor properties embedded in a CpG-enriched region of the genome such as Maspin or METTL3 [55,56]. The use of this new tool will be useful not only for the understanding of the epigenetic modifications in an endogenous biological context but also for the potential cancer therapies based on these findings.

Studies have shown that across the gastric precancerous cascade, *RPRM* becomes increasingly hypermethylated, in association with loss of protein expression. These findings are particularly related with the transition from intestinal metaplasia to dysplasia and/or gastric cancer [57]. Consequently, no differences in methylation levels have been found between paired tumor and non-tumor adjacent cells [3]. Taken together, loss of expression and/or methylation of *RPRM* could be proposed as a late event in the gastric precancerous cascade. Methylation of the *RPRM* promoter region is associated with the infection of *Helicobacter pylori* particularly to cytotoxin-associated gene A (CagA) strains [58]. Accordingly, methylation of *RPRM* promoter region has been proposed as a tissue biomarker for the evaluation of *H. pylori* eradication [59].

Accordingly, with this line of evidence, follow-up studies examining DNA methylation levels of *RPRM* on the longitudinal progression of the gastric precancerous lesions after *H. pylori* eradication, have revealed an increasing level of the DNA methylation six-years prior the progression of gastric lesions [60]. Interestingly, these changes were independent of the effect of the duration of *H. pylori* infection and other clinical parameters [60].

Since *RPRML* is also located within a CpG-enriched region of the genome, as has been well documented in the case of *RPRM* [3,35,48,49] and also correlates with transcriptional silencing [3,32], the evaluation of *RPRML* methylation may yield similar findings to that of *RPRM*. To assess this issue, an exploratory in silico analysis of RNAseq expression data from paired gastric adenocarcinomas and non-tumor adjacent mucosa from The Cancer Genome Atlas (TCGA) database [0] is in progress. In addition, in vitro gain/loss of function experiments focused on evaluation of tumorigenic or tumor suppressive effects may provide insights on the role of *RPRML* in cancer. 

## 7. Methylated *RPRM* Cell-Free DNA as a Potential Non-Invasive Biomarker in Gastric Cancer

The discovery of methylated cell-free DNA in body fluids has expanded the translational applications of DNA methylation of cancer-related genes [61,62]. Due to its relatively stable nature and availability, DNA methylation can be easily detected in serum, plasma and a variety of body fluids. Thus, the assessment of DNA methylation through cell-free DNA or liquid biopsy approaches has been proposed as a candidate for the diagnosis and management of cancer [63].

Our group was the first to propose the assessment of methylated cell-free DNA of the *RPRM* promoter gene region for non-invasive detection of gastric cancer with a sensitivity of 95.35% [95% CI: 84.19–99.43%] and specificity of 90.32% [95% CI: 74.25–97.96%] [48]. These values achieved the highest OR (OR = 191.33 [95% CI = 30.01, 1220.01]) in a comprehensive meta-analyses undertaken by Sapari el al. [64] after consolidation of 132 case-controls studies for potential biomarkers based on methylated DNA in gastric cancer. This approach has subsequently been extended to the detection of precancerous lesions [32,57,65]. Therefore, clinical trials addressing the role of *RPRM* as a non-invasive biomarker in gastric cancer should be performed.

## 8. Unanswered Questions and the *RPRM* Gene Family

There are many biological questions still to be answered regarding *RPRM* gene family. Protein structure and the biological relevance of post-translational modifications such as glycosylation, phosphorylation and sumoylation should be assessed (Figure 1). Furthermore, the regulation of expression and the translational applications of the *RPRM* gene family in cancer medicine need to be further elucidated, along with a potential role in other pathologies [4]. Originally described as a p53-dependent cell cycle arrest mediator (Figure 4), yet the functions and mechanisms by which RPRM acts still remain unknown. Evolutionary studies, developmental genetics and molecular biology approaches will prove useful tools in determining the origin and function of this single exon and intronless *RPRM* gene family [7,66]. The evaluation of different and complementary lines of evidence such as (1) selective pressures; (2) phenotypical variations as a consequence of loss- or gain-of-function; and (3) pre- and post-transcriptional and translational variations may deliver a better understanding of this gene family. From an evolutionary perspective, comparative genomics and phylogenetic analyses may provide evidence of co-evolution and potential relationships between *RPRM* and other gene families, and thus uncover new signaling partners. Questions regarding a “de novo” origin or retrotranscription of mRNA followed by insertion of the resulting DNA copy into the genome should be addressed to clarify the origin and evolution of the *RPRM* single exon gene family. Using molecular and genetic approaches, which rely on DNA sequence or RNA/protein expression alterations, it may be possible to establish the pathophysiological role of the *RPRM* genes in the regulation of cellular functions. Mutation of *RPRM* family genes will be useful to identify phenotypic defects generated by *RPRM* loss-of-function as well as to dissect the role of *RPRM* in developmental processes. 

**Figure 5:**
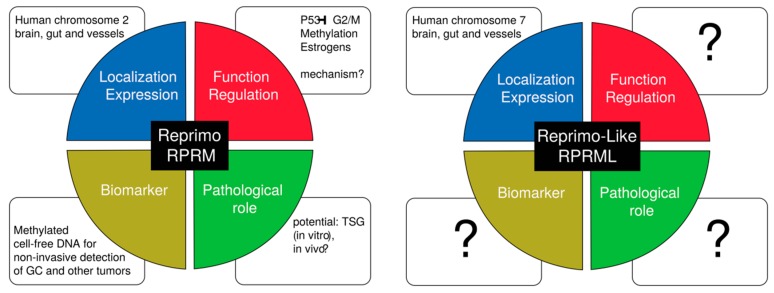
Unanswered questions in *RPRM* gene family. RPRM is as a p53-induced protein which induces cell cycle arrest at the G2/M checkpoint [4], through an unknown mechanism. Recently, *RPRM* genes have been shown to be expressed during brain, gut and blood vessel development [2]. Additional functions for *RPRM* include its role as a potential tumor suppressor gene (TSG), and a biomarker—through the assessment of the methylation status of its promoter region—for non-invasive detection of gastric cancer and other tumors [48,62]. Much like *RPRM*, *RPRML* is also expressed during embryonic development [2], but its role in physiological processes has never been investigated.

The function and role of the *RPRM* genes in gastric carcinogenesis should be expanded to incorporate *RPRML* (Figure 5); as to date, only the human *RPRM* gene has been examined. Importantly, the unique expression pattern of *rprml* suggests that this is a functional gene and will hopefully initiate studies into its presence and function in human gastric physiology and cancer tissues. 

Although the ectopic expression of *RPRM* induces cell cycle arrest and apoptosis in vitro [4,34,55], unanswered questions still remain as to how transcriptional silencing of *RPRM* predisposes tissues to gastric cancer development. In multiple tumors, DNA methylation is the most common epigenetic mechanism of loss-of-expression of *RPRM* [62]. In fact, loss-of-expression by DNA methylation of the *RPRM* promoter region it is an indicator of cancer progression and the continuous use of demethylating agents such 5-aza -cytidine can restore *RPRM* gene function and dampen indicators of tumor progression [3]. In this scenario, more specific approaches to re-activate *RPRM* expression, for example through CRISPR/dCas9-based artificial transcription factors, may open the door to new translational opportunities in cancer therapeutics.

## 9. Concluding Remarks

The role of the *RPRM* gene family in vertebrate physiology and disease is still a bourgeoning field. A comprehensive characterization of the genetic interactions, signaling pathways, protein modifications and regulatory mechanisms of the *RPRM* gene family may shed light on its role in both physiological and oncological processes. *RPRM* is known to play a role in tumors of the stomach and has translational applications in the monitoring and treatment of disease. Whether other members of this gene family also have tumor suppressor properties or play relevant roles in other pathologies, remains a key question in need of further research. In this scenario, this family of single exon genes could lead to the discovery of novel biomarkers and therapeutic targets, together with the development of new drugs and clinical applications. The coming years should bring forth active investigation to help understand, define and utilize the *RPRM* gene family.
